# Patient-Centered Lupus Erythematosus Mobile Apps: Systematic Search and Cross-Sectional Evaluation by Patients and Physicians

**DOI:** 10.2196/73019

**Published:** 2026-05-29

**Authors:** Tassilo Dege, Antonia Ullmann, Caroline Glatzel, Janik Fleißner, Vanessa Borst, Patrick-Pascal Strunz, Marc Schmalzing, Matthias Goebeler, Astrid Schmieder

**Affiliations:** 1 Department of Dermatology, Venereology and Allergology, Universitätsklinikum Würzburg, Josef-Schneider-Str. 2, Würzburg, 97080, Germany, 49 931-2010; 2 Department of Computer Science, University of Würzburg, Würzburg, Germany; 3 Department of Rheumatology, Universitätsklinikum Würzburg, Würzburg, Germany

**Keywords:** lupus erythematosus, systemic lupus erythematosus, cutaneous lupus erythematosus, mobile applications, evaluation, mental health, mars, sus, ati, teledermatology, disease management, health app, skin, eHealth, mHealth, telemedicine

## Abstract

**Background:**

Lupus erythematosus (LE) is a chronic autoimmune disease that significantly impacts patients’ quality of life. Photosensitivity is a key impairment that severely limits the quality of life, especially in cutaneous lupus erythematosus (CLE), where exposure to sunlight can lead to rashes, exacerbations, and pain. In systemic lupus erythematosus (SLE), other manifestations such as joint pain, fatigue, and organ damage may contribute to decreased physical function and emotional distress. Mobile health apps (MHA) offer potential support for comprehensive disease management for the symptoms mentioned above. However, there is a lack of systematic analysis of available lupus management apps.

**Objective:**

This study aims to systematically identify publicly available German or English MHA for lupus management as well as to assess their quality by surveying both patients and physicians.

**Methods:**

A systematic search and assessment of German or English mobile apps for patients with lupus, available in the Google Play Store and Apple App Store, was conducted independently by two reviewers. The two apps that met all relevant criteria were then reviewed independently by seven physicians using the German Mobile Application Rating Scale (MARS) and the System Usability Scale (SUS). Subsequently, they were reviewed by five patients (three with SLE and two with CLE), using the user version of MARS (uMARS) and SUS. Additionally, the Affinity for Technology Interaction (ATI) scale was collected from both patients and physicians to evaluate the technical affinity in both groups.

**Results:**

In total, 29 apps were available on the Apple Store and 26 on the Google Store, with 18 apps being present and downloadable on both platforms. Of the 18 apps, 16 were excluded because they did not meet the inclusion and exclusion criteria. Only two apps, *Lupus Log* and *Lupus Minder* met all the required criteria and were included in the study. The mean MARS scores varied from 2.61/5 to 4.17/5 and mean SUS from 17.5/100 to 100/100 between physicians. The app with the highest mean overall MARS score was *Lupus Log*, which was rated with 3.91/5 on average by the physicians. Patients evaluated the app with a comparably mean uMARS score (3.95/5). Technical affinity, objectified by ATI, was higher in patients than physicians (3.9 vs 3.68).

**Conclusions:**

Systematic identification and evaluation showed high-quality apps for patient-centered lupus MHA as indicated by MARS and uMARS scores greater than 3 for both *Lupus Log* and *Lupus Minder*.

## Introduction

### Background

Lupus erythematosus (LE) is a chronic autoimmune disorder with great heterogeneity of symptoms. It can affect multiple organs, including skin, kidney, heart, and the nervous system [1]. In cutaneous lupus erythematosus (CLE), inflammation is typically confined to the skin and does not affect internal organs. CLE is often worsened or even triggered by sun exposure [2]. Systemic lupus erythematosus (SLE) can present with many clinical symptoms of varying severity, including lupus nephritis, which can lead to kidney failure if left untreated. Therefore, SLE patients are treated with systemic corticosteroids and other immunosuppressants or immunomodulators, which can again cause side effects and result in additional morbidity. In contrast, for CLE, topical treatments are often sufficient to relieve symptoms [3].

Due to chronic inflammation, patients experience a variety of physical limitations, including joint pain, fatigue, sleep disorders, photosensitivity as well as cardiovascular and pulmonary symptoms. Mental and emotional health is further impaired with patients showing high prevalence of depression, anxiety, and cognitive disorders [4]. Furthermore, SLE can also directly affect the central nervous system causing a wide variety of psychiatric symptoms [5]. Some patients experience a so called “lupus fog” with memory loss and difficulty concentrating, which further negatively impacts daily life and social interactions [6]. Corticosteroids and immunosuppressive drugs can cause weight gain, diabetes, osteoporosis, and increased risk of infection that are detrimental to patients’ quality of life [7].

In people living with LE, symptoms such as chronic fatigue, joint pain, and cognitive difficulties often occur unnoticed by others, making it hard for patients to feel understood and to receive empathy. This invisibility can lead to several emotional and social struggles such as frustration and a feeling of stigmatization [8]. The unpredictability of lupus flare-ups makes this disease more challenging. Even when the disease appears to be controlled, symptoms can suddenly return and cause fear and anxiety [9].

Since patients with lupus often experience fluctuating symptoms, it can complicate treatment adherence and monitoring. Therefore, mobile health apps (MHA) present an opportunity to support patients in managing their condition by providing accessible information, symptom tracking, and direct communication with health care providers [10]. However, there is a lack of robust evidence regarding the effectiveness of these apps for lupus patients, and high-quality studies are essential to assess their impact [11]. To date, comprehensive reviews of MHA for lupus management have been scarce [12], as they were restricted to evaluations conducted exclusively by health care professionals (with small reviewer samples) and without incorporating patient perspectives. To complement existing reviews, a comparative analysis using validated scoring systems and involving both health professionals and patients is needed to generate more robust data on the usability and quality of MHAs for lupus self-management. The Affinity for Technology Interaction (ATI) scale provides a reliable method for quantifying an individual’s technology affinity, providing important context for interpreting their app evaluations [13]. To subjectively assess app quality, the Mobile App Rating Scale (MARS) was developed, evaluating aspects such as engagement, functionality, esthetics, and information [14-16]. MARS has been applied in studies evaluating mobile apps related to various chronic diseases, including chronic wounds [17]. A user version (uMARS) allows app quality to be assessed from the patient perspective [16]. The System Usability Scale (SUS), a 10-item questionnaire, has been used broadly to assess the usability of systems, including mobile apps for conditions like dementia, depression, pediatric obesity, and smoking cessation [18].

### Aim of This Study

This study aims to systematically identify and assess mobile apps available for lupus management. By using validated tools, such as the MARS and the SUS, this research seeks to provide a comprehensive evaluation of these apps from both professional and CLE as well as SLE patient perspectives.

## Methods

### Study Design and Setting

This was a cross-sectional observational study conducted at the Department of Dermatology, University Hospital Würzburg, Germany. The study involved a systematic search and comparative evaluation of MHAs for patients with LE by both patients and physicians.

### Participants

Physicians were dermatologists involved in direct lupus patient care. Patients were adults (≥18 y) with a confirmed diagnosis of cutaneous or systemic lupus erythematosus, identified during routine outpatient visits.

### Ethical Considerations

This study was conducted in accordance with ethical guidelines and standards, with approval from the ethics committee at the University of Würzburg (reference number 2022062501). All participants provided written informed consent. No personal identifiers were collected on the forms, and responses were manually entered by the study team. All data were deidentified at the point of analysis. No financial or material compensation was provided to participants for taking part in the study.

### App Identification and Selection

Two independent reviewers systematically searched the Apple App Store and the Google Play Store in June and July of 2023 to identify patient-centered mobile health applications related LE. The search terms used were: “lupus,”, “lupus erythematosus,” “lupus erythematodes,” “systemic lupus,” “cutaneous lupus”. German and English apps were included in order to reflect the languages most used by the study population, and to ensure relevance to the target user group. To guarantee cross-platform usability and broader accessibility, only apps available in both app stores were considered. Additionally, it was required that apps were specifically designed for patients with LE, were free of charge, did not require user registration or community interaction, and did not contain advertisements. We excluded apps with advertisements to prevent disruptions in users’ experiences and to avoid the confounding effects advertisements can have on usability [19]. Advertisements can frustrate users, reduce the clarity and intuitiveness of an app’s interface, and make it difficult to compare apps’ performance or users’ experiences [20]. As part of the inclusion criteria, only apps with a publicly accessible privacy policy and evidence of basic data protection measures were included. This ensured alignment with good practice guidelines for mobile health evaluations.

### Data Extraction and App Characteristics

The following information available in the app stores and on app websites was collected

- App name
- Target consumer group (eg, patients, medical personnel)
- Cost
- Platform
- Advertisement
- Features
- Search term used to identify the app

### Evaluation

#### Mobile Application Rating Scale

The quality of the identified and included apps was evaluated by seven physicians using the MARS, which assesses the four domains: engagement, functionality, esthetics, and information. Each item is rated on a 5-point Likert scale, where 1 indicates “inadequate” and 5 indicates “excellent” performance. Higher scores reflect better app quality. The overall MARS score is calculated as the mean of the subdomain scores, with a maximum possible score of 5; a score above 3 is considered acceptable or good app quality. The apps were then further evaluated by five patients (three with systemic lupus erythematosus and two with cutaneous lupus erythematosus) using the user version of the scale (ie, uMARS), which uses the same scoring logic and dimensions but is adapted for the user perspective.

#### System Usability Scale

In addition, the SUS was applied to assess overall usability. SUS consists of 10 items rated on a 5-point Likert scale, with the total score converted to a 0‐100 scale. Higher SUS scores indicate better usability. A SUS score ≥70 is generally considered acceptable, scores between 50 and 70 indicate marginal usability, and scores below 50 are interpreted as poor usability. Including both MARS and SUS allowed for a comprehensive assessment of app quality and usability from both the health care professional and the patient viewpoint.

#### Affinity for Technology Interaction

The ATI scale was used to gather information on the physicians‘ and patients‘ technical affinity. ATI consists of 9 items rated on a 6-point Likert scale, resulting in a score range from 1 (very low affinity) to 6 (very high affinity). Higher scores indicate a greater interest in interacting with technology. A score above three reflects average or moderate technology affinity, while a score above four is typically interpreted as high. Assessing ATI in both user groups provided context for interpreting their app ratings, particularly in terms of potential differences in expectations, familiarity, and digital literacy.

### Evaluation Procedures

Each physician and patient received access to the same apps and tested them on their own smartphones or tablets (iOS or Android). Participants were instructed to test each app for at least ten minutes, explore all relevant features, and assess its usability, content, and overall functionality. To assist participants in understanding the rating process, an optional explanatory video describing the MARS/uMARS tool was made available via a QR code. Following app testing, participants completed the appropriate paper-based evaluation tools. All questionnaires were filled out on paper and collected by the study team for manual data entry and analysis.

### Statistical Analysis

Descriptive statistics were used to summarize app quality scores and demographic data. The Mann-Whitney *U* test was used to compare scores between patients and physicians (eg, MARS vs. uMARS, SUS, ATI). The Wilcoxon signed-rank test was applied to compare differences between apps. *P* values below .05 were considered significant. The data analysis was performed using Python, with libraries such as Pandas, SciPy [21], NumPy [22], Matplotlib, and Seaborn [23].

## Results

### App Selection

**Figure 1.**
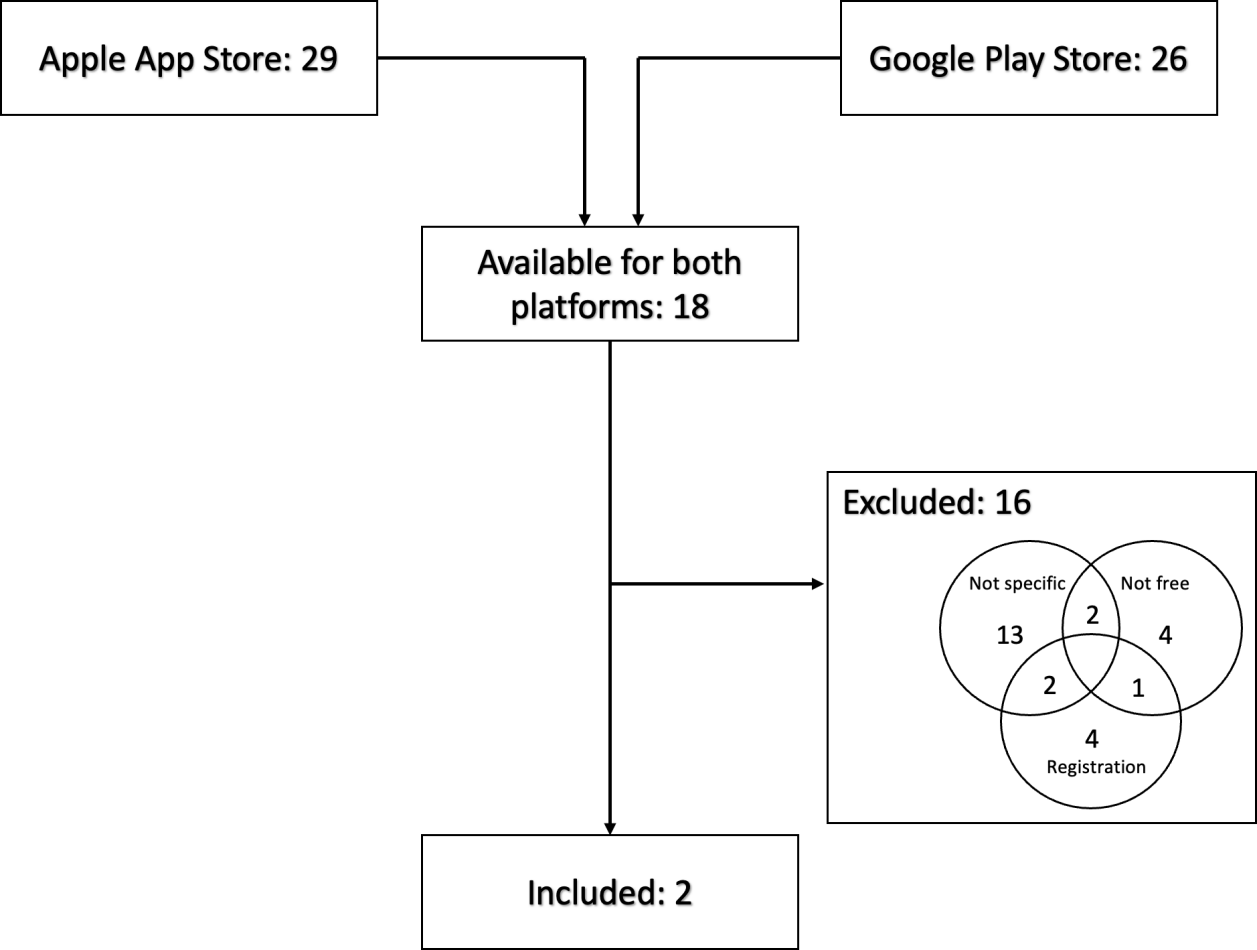
Flowchart illustrating the screening process for identifying suitable mobile apps.

In total, 37 potentially relevant mobile health apps targeting lupus patients were initially identified through the app store search. These apps were then reviewed independently by two authors to assess eligibility according to predefined inclusion and exclusion criteria. Of the 37, 18 apps were available in both the Apple App Store and Google Play Store (see Figure 1). Of these 18 apps, 10 were targeted at patients. Strict criteria were established for app inclusion: the apps needed to be specifically designed for LE, free of charge, and did not require registration/community connections, or advertising. Of the 18 apps, 13 were excluded because they were not specifically designed for LE. Additionally, 4 apps required payment after download or for full access, and 4 apps asked for registration, included ads, or had community connections. Some apps met multiple exclusion criteria. Ultimately, only 2 apps, *Lupus Log* and *Lupus Minder*, met all the required criteria and were included in the study.

### Evaluation of App Quality

A total of two apps —*Lupus Log* and *Lupus Minder* —met all inclusion and exclusion criteria and were evaluated by 7 physicians. *Lupus Log* and *Lupus Minder* are designed specifically for lupus patients, offering features tailored to disease management and symptom tracking. Both apps provided a user-friendly interface and allow for the creation of a personalized medication plan. *Lupus Minder* features a structured design with an intuitive layout. It offers general lupus information and a planner for medical appointments, which can be printed or exported. A daily symptom tracker is available, but it only includes a free-text field without predefined options. The app ensures data security through password protection. *Lupus Log* stood out for its well-structured design and includes a real-time, location-based UV index. Its daily symptom tracker allows users to select predefined symptoms, add custom categories (eg, fatigue, lesions, ulcers), and to attach notes or photos. The app also provides lupus-related tips, news updates, and access to personal health statistics. Additionally, push notifications can remind users to complete the symptom tracker, though medication reminders are not included.

The mean MARS scores (and SDs) of the physicians, including subcategory scores and SUS scores, are provided in Table 1, while the corresponding uMARS and SUS results from patient evaluations are shown in Table 2.

**Table 1.** MARS^a^ and SUS^b^ scores of the *Lupus Log* and *Lupus Minder* app, as assessed by 7 physicians.

Scores	Lupus Log	Lupus Minder
MARS, mean (SD)	3.91 (0.42)	3.29 (0.38)
A: Engagement score, mean (SD)	3.69 (0.58)	2.86 (0.65)
B: Functionality score, mean (SD)	4.46 (0.37)	3.75 (0.46)
C: Esthetics score, mean (SD)	4.19 (0.72)	3.24 (0.66)
D: Information score, mean (SD)	3.48 (0.65)	3.43 (0.26)
SUS, mean (SD)	87.14 (4.43)	65.36 (27.59)

a MARS: Mobile Application Rating Scale.

b SUS: System Usability Score.:

**Table 2.** uMARS^a^ and SUS^b^ scores of the *Lupus Log* and *Lupus Minder* app, as assessed by 5 patients.

Scores	Lupus log	Lupus minder
uMARS, mean (SD)	3.95 (0.62)	3.32 (0.52)
A: Engagement score, mean (SD)	3 (0.94)	2.4 (0.52)
B: Functionality score, mean (SD)	4.35 (0.93)	3.8 (1)
C: Esthetics score, mean (SD)	4.41 (0.44)	3.74 (0.91)
D: Information score, mean (SD)	4 (0.5)	3.56 (0.43)
SUS	76 (29.18)	49.38 (34.18)

a uMARS: User version of Mobile Application Rating Scale.

b SUS: System Usability Score.

*Lupus Log* had the highest mean MARS (mean 3.91, SD 0.42 out of 5) and mean uMARS (mean 3.95, SD 0.62 out of 5). When Wilcoxon signed-rank test was applied *Lupus Log* had a significantly higher MARS score (*P=.*03*)* but not significantly higher uMARS scores (*P=*.125), possibly due to small size (see detailed information on demographics, uMARS, SUS and ATI scores of CLE and SLE patients in Multimedia Appendix 1). *Lupus Minder* had a lower mean MARS (mean 3.29, SD 0.38) and mean uMARS (mean 3.31, SD 0.52 out of 5). Mean SUS by physicians and patients scores were 87% and 76% for *Lupus Minder* and 65% and 49% for *Lupus Log,* respectively.

### Comparative Analysis of Patients and Physicians Data

*Lupus Log* MARS (see Table 1) and uMARS (see Table 2) were almost identical and differences were not statistically significant (*P=*.76). In the subcategory engagement and information, mean scores differed most. Patients rated engagement with 3.0 lower than physicians 3.69 (*P=*.16). In contrast, information was rated higher by patients with a mean score of 4.0 than by physicians with a mean score of 3.48 (*P=*.19).

*Lupus Minder’s* MARS (see Table 1) score and uMARS (see Table 2) were almost identical (*P=.*70). In the subcategory engagement and esthetics, mean scores differed most. Patients rated engagement with 2.4 lower than physicians with 2.86 (*P=*.30). In contrast, esthetics was rated higher by patients with a mean score of 3.74 than by physicians with a mean score of 3.24 (*P=*.40).

### Evaluation of Technical Affinity

**Figure 2.**
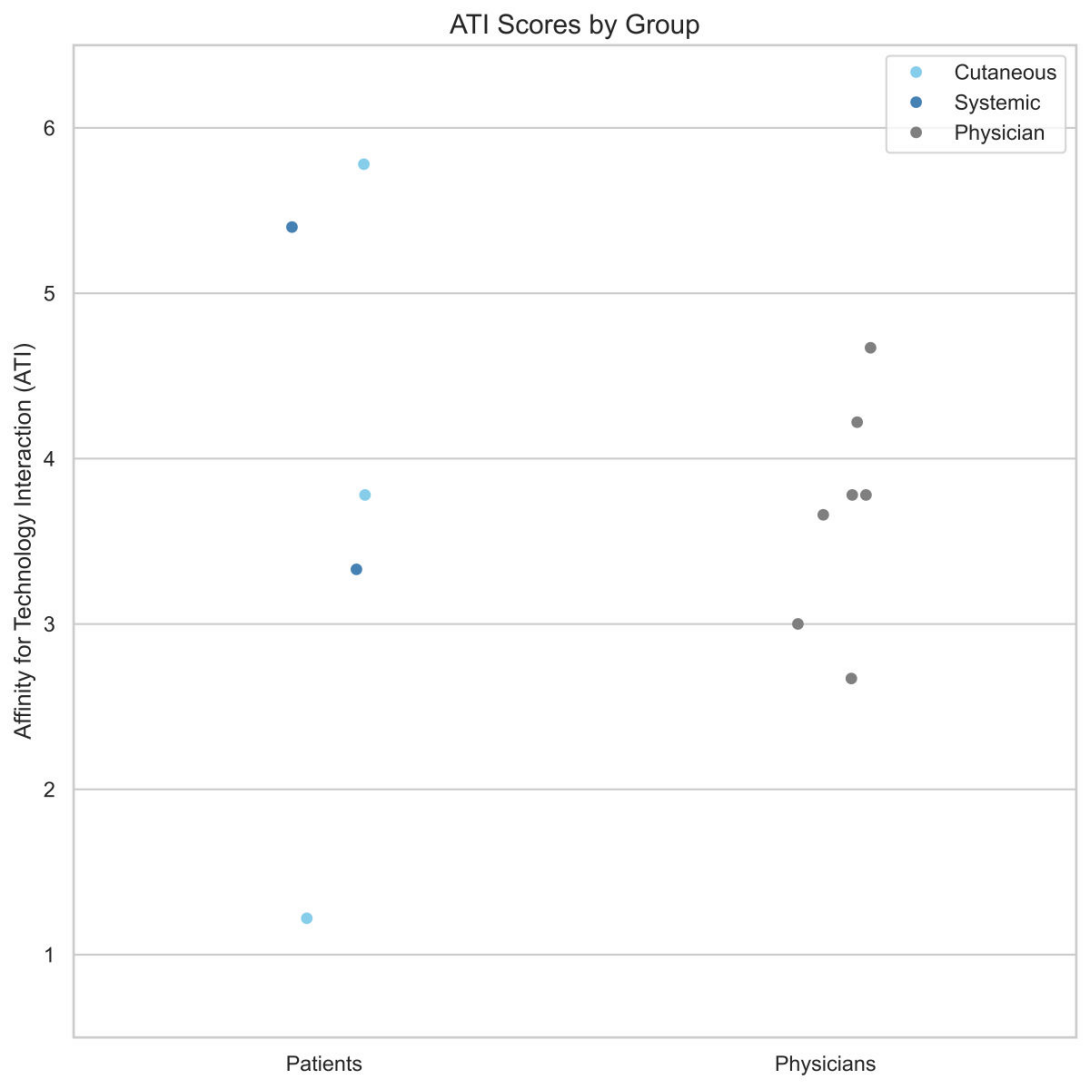
Individual ATI scores (range: 1‐6) for patients with cutaneous (light blue) or systemic (dark blue) lupus erythematosus and physicians (gray). Patients showed slightly higher scores than physicians, though not statistically significant (*P=*.74, Mann-Whitney *U* test).

The physician group consisted of 5 females and 2 males, with an age range of 27‐42 years (median: 30 y). The patient group included 3 patients with systemic lupus erythematosus (SLE) and 2 with cutaneous lupus erythematosus (CLE). Four patients were female, and one was male, with ages ranging from 35 to 67 years (median: 52 y). ATI scores ranged from 1.22 to 5.78 out of 6. An ATI score of >3 indicates average technology affinity, and a score of >4 indicates high technology affinity. Technical affinity was lower in physicians than in patients (3.68, SD 0.68 vs 3.9, SD 1.82) but this difference was not statistically significant (*P*=.74). A visualization of ATI scores in both groups is shown in Figure 2.

## Discussion

### Principal Findings

In this study, we first conducted a systematic search to identify smartphone apps specifically designed for patients with LE, following approaches used in previous evaluations of mobile health apps [24-26]. Among the included apps, *Lupus Log* received a significantly higher MARS score than *Lupus Minder* (*P*=.03), indicating a clear preference among physicians regarding overall app quality. However, no statistically significant differences were observed in the uMARS scores among patients (*P=.12*), nor between patients and physicians across most subdomains, possibly due to the small sample size and the limited power to detect subtle differences. Although several subgroup comparisons (eg, ATI scores between groups, SUS scores between apps) showed meaningful trends, they did not reach statistical significance.

High agreement among assessors, even for subjective measures, is an encouraging indicator of the reliability of the assessment. While the Enlight [27] framework is another feasible and validated tool for digital health app evaluation, MARS and uMARS were chosen for this study due to its established use in comparative assessments by both health professionals and patients [25], aligning with the study’s focus on multi-stakeholder perspectives.

This study identified a limited number of high-quality lupus management apps that met our strict inclusion criteria. Both *Lupus Log* and *Lupus Minder* received decent ratings when rated by raters who can be considered affine to technology based on mean ATI scores greater than 3 for physicians and patients. With a mean MARS score of 3.91 (SD 0.42), *Lupus Log* was rated highest by physicians. Likewise, it achieved a mean uMARS score of 3.95 (SD 0.62). In the subcategory ‚information‘, *Lupus Log* was rated higher by patients overall and in the subcategory ‘engagement,’ possibly due to offering additional information on UV index.

Previous studies have assessed the quality of lupus-related mobile health apps but were limited to evaluations conducted exclusively by healthcare professionals, often with only two raters per app. Ramasamy et al [11] evaluated 19 apps for systemic lupus erythematosus (SLE) and lupus nephritis, reporting that *Lupus Minder* received an overall MARS score of 2.96, with domain-specific scores of 2.9 (engagement), 3.5 (functionality), 3.1 (esthetics), and 2.35 (information). In a separate systematic review, Dantas et al [12] evaluated *Lupus Log*, which scored 2.7 overall, including 3.3 (engagement), 3.2 (functionality), 2.3 (esthetics), and 2.1 (information). While informative, both studies relied on assessments from only two health care professionals per app, without incorporating patient perspectives. In contrast, our study included a broader group of raters—seven physicians and five patients—and uniquely combines both clinical and user-centered evaluations. This dual-stakeholder approach provides a more comprehensive evaluation of app quality and usability. Compared to the previous reviews by Dantas et al and Ramasamy et al, which identified a larger number of lupus apps, our study applied stricter inclusion criteria focused on real-world usability; we included only apps that were free, available on both major platforms, and did not require registration or contain advertising. In contrast, both prior reviews allowed paid apps, iOS-only apps, and apps with limited accessibility.

The episodic nature of lupus makes assessing disease activity and severity challenging, as flare-ups are unpredictable and often go unobserved by physicians. Current evaluations rely on patients’ memories, subjective reports, and occasional photos, which can lead to misjudgments and suboptimal treatment. Regular monitoring patient-reported outcome measures is therefore essential, as recommended by lupus management guidelines [28], which advocate for validated tools like LupusQoL [29] and SLAQ (Systemic Lupus Activity Questionnaire) [30] to assess disease control. A mobile health app can simplify this process by enabling continuous tracking of symptoms, severity, and flare-ups while providing an overview for both patients and physicians. This improves support, builds confidence, and enhances treatment precision for physicians and patients.

However, patient involvement in the app development process remains limited [31]. As the primary end users, patients should play a more active role in the co-design and development process to ensure usability, relevance, and engagement [32,33] Both patient-centered apps evaluated in this study, provided a diary function which serves both physicians and patients. While no prior studies have evaluated mobile apps for lupus erythematosus using both MARS and uMARS, comparisons with evaluations in other disease areas, such as mental health, are informative. A recent study using MARS to assess French-language mental health apps [34] reported a top MARS score of 3.85, which is comparable to the scores observed in our study. These findings suggest that the quality of our top-rated apps is on par with apps in better-studied domains, such as mental health.

### Limitations

The study’s limitations include a small sample size and the subjective nature of the evaluation tools. Future research should involve larger patient cohorts and consider randomized controlled trials to assess app effectiveness more robustly over a longer period. Other app platforms (eg, Huawei AppGallery, Samsung Galaxy Store, Windows Phone Store and Blackberry World) were excluded from the search to focus on the most used platforms—Apple App Store and Google Play Store—which dominate the mobile health app market in Western countries. This exclusion is acknowledged as a limitation of the study. By excluding apps with advertisements, some potentially relevant apps may have been omitted. This narrower inclusion strategy, while intentionally designed, likely contributed to the lower number of included apps compared to earlier reviews.

### Conclusions

*Lupus Log* and *Lupus Minder* offer practical tools for tracking disease activity in both CLE and SLE. These apps have received positive feedback from patients and physicians, supporting disease control and promoting patient self-management. Their acceptance among patients highlights the need for further studies to evaluate long-term use and adapt the apps to meet the needs of both groups. Involving patients in the app design process can enhance usability and relevance, ultimately leading to better disease management outcomes.

## Supplementary material

10.2196/73019 Multimedia Appendix 1 Patient (A) and physician (B) characteristics (age and sex) and the Affinity for Technology Interaction scale, System Usability Scale, Mobile App Rating Scale (MARS), and user version of the MARS scores (including subcategory scores) of all 5 patients and 7 physicians who participated.

## Abbreviations

ATI: affinity for technology interaction
CLE: cutaneous lupus erythematosus
LE: lupus erythematosus
MARS: Mobile Application Rating Scale
MHA: Mobile health apps
SLAQ: Systemic Lupus Activity Questionnaire
SLE: systemic lupus erythematosus
SUS: System Usability Scale
uMARS: user version of Mobile Application Rating Scale
