# Supplementary material for: Patient-Centered Lupus Erythematosus Mobile Apps: Systematic Search and Cross-Sectional Evaluation by Patients and Physicians
Source: JMIR Mhealth Uhealth. 2026 May 29;14:e73019. doi: 10.2196/73019 (PMC13221119; doi:10.2196/73019)
Supplement: Multimedia Appendix 1 [file mhealth-v14-e73019-s001.doc]

|  |  |  |  |  |  | **Lupus Log** | | | | | | | | **Lupus Minder** | | | | | | | |
| --- | --- | --- | --- | --- | --- | --- | --- | --- | --- | --- | --- | --- | --- | --- | --- | --- | --- | --- | --- | --- | --- |
| **Group** | **ID** | **Sex** | **Age** | **Platform** | **ATI** | **A** | **B** | **C** | **D** | **E** | **F** | **MARS** | **SUS** | **A** | **B** | **C** | **D** | **E** | **F** | **MARS** | **SUS** |
| SLE | 1 | f | 41 | Android | 5,78 | 2,6 | 4,5 | 4,7 | 3,75 | 2,5 | 2,5 | 3,9 | 75% | 2,6 | 5 | 4,3 | 3,5 | 2,75 | 2,83 | 3,85 | 100% |
| SLE | 2 | f | 58 | Android | 1,22 | 1,6 | 2,75 | 4 | 3,33 | 3 | 4,17 | 2,92 | 27,50% | n/a | n/a | n/a | n/a | n/a | n/a | n/a | n/a |
| SLE | 3 | f | 52 | Android | 3,78 | 3,6 | 4,5 | 4 | 4 | 3,25 | 3,67 | 4,03 | 80% | 1,8 | 3,75 | 3,33 | 3 | 2,25 | 2,33 | 2,97 | 30% |
| CLE | 4 | f | 35 | iOS | 3,33 | 3,2 | 5 | 5 | 4,5 | 4 | 3,16 | 4,43 | 100% | 2,2 | 2,5 | 2,67 | 3,75 | 1,25 | 3,33 | 2,78 | 27,50% |
| CLE | 5 | m | 67 | iOS | 5,4 | 4 | 5 | 4,33 | 4,5 | 3,75 | 5 | 4,46 | 97,50% | 3 | 4 | 4,67 | 4 | 2 | 2,33 | 3,67 | 40% |

**A**

|  |  |  |  |  | **Lupus Log** | | | | | | | | | **Lupus Minder** | | | | | | | | |
| --- | --- | --- | --- | --- | --- | --- | --- | --- | --- | --- | --- | --- | --- | --- | --- | --- | --- | --- | --- | --- | --- | --- |
| **ID** | **Sex** | **Age** | **Platform** | **ATI** | **A** | **B** | **C** | **D** | **E** | **F** | **MARS** | **SUS** | **A** | | **B** | **C** | **D** | **E** | **F** | **MARS** | **SUS** |  |
| 1 | f | 42 | iOS | 3.66 | 4.2 | 5 | 5 | 3.75 | 3.75 | 4.17 | 4.49 | 92.50% | 1.8 | | 3 | 2.67 | 3 | 1.25 | 1.83 | 2.61 | 17.50% |  |
| 2 | f | 25 | iOS | 2.67 | 3 | 4.75 | 5 | 2.75 | 2.5 | 3.83 | 3.88 | 80% | 2.4 | | 3.75 | 2.33 | 3.25 | 1.5 | 2.83 | 2.93 | 80% |  |
| 3 | f | 29 | iOS | 3.78 | 4 | 4.25 | 4.67 | 4.25 | 3 | 3.83 | 4.29 | 90% | 3.4 | | 3.25 | 4.33 | 3.5 | 2.5 | 3.33 | 3.62 | 77.50% |  |
| 4 | f | 32 | iOS | 4.22 | 2.8 | 4 | 3.33 | 3.33 | 2.5 | 3 | 3.37 | 82.50% | 2.8 | | 4 | 3.33 | 3.33 | 2.5 | 2.83 | 3.37 | 82.50% |  |
| 5 | m | 29 | iOS | 3 | 4.2 | 4.75 | 4 | 3.75 | 3.75 | 4.33 | 4.18 | 90% | 2.8 | | 4 | 3.33 | 3.5 | 2 | 2.67 | 3.41 | 90% |  |
| 6 | m | 30 | iOS | 4.67 | 4 | 4.25 | 4 | 2.5 | 3 | 3.83 | 3.69 | 87.50% | 3 | | 4.25 | 3.67 | 3.75 | 2.75 | 3.67 | 3.67 | 75% |  |
| 7 | f | 27 | iOS | 3.78 | 3.6 | 4.25 | 3.33 | 4 | 2.25 | 3.25 | 3.48 | 87.50% | 3.8 | | 4 | 3 | 3.66 | 2.75 | 3 | 3.44 | 35% |  |

**B**
